# Salidroside Protects Against Simazine-Induced Neurotoxicity by Activating *PINK1/Parkin* Mitophagy

**DOI:** 10.3390/ijms27104242

**Published:** 2026-05-10

**Authors:** Xueting Li, Yi Xiang, Jiaqi Li, Hewei Song, Chunlong Zhao, Baixiang Li

**Affiliations:** 1 Department of Hygienic Toxicology, School of Public Health, Harbin Medical University, Harbin 150081, China; lxting@hrbmu.edu.cn (X.L.); 2024020249@hrbmu.edu.cn (Y.X.); 2024020205@hrbmu.edu.cn (J.L.); 2024020210@hrbmu.edu.cn (H.S.); 2024020203@hrbmu.edu.cn (C.Z.); 2 Key Laboratory of Precision Nutrition and Health, Ministry of Education, Harbin Medical University, Harbin 150081, China

**Keywords:** salidroside, simazine, SH-SY5Y, neuroprotection, mitophagy, apoptosis

## Abstract

Simazine (SIM), a triazine herbicide and potential environmental risk factor, has been associated with neurotoxicity; however, the underlying mechanisms remain poorly characterized. Salidroside (SAL), a natural antioxidant with mitochondrial protective properties, has been reported to alleviate SIM-induced neuronal injury. Using an integrated strategy combining network toxicology and network pharmacology with experimental validation, this study systematically investigated the neurotoxic mechanisms of SIM and the neuroprotective effects of SAL. Bioinformatics analyses revealed that SIM- and SAL-related targets were significantly enriched in apoptosis- and autophagy-associated pathways. In vitro experiments demonstrated that SIM induced mitochondrial structural damage, metabolic dysfunction, and dopaminergic neuron-like SH-SY5Y cells apoptosis by inhibiting *PINK1/Parkin*-mediated mitophagy. Conversely, SAL effectively protected SH-SY5Y cells against SIM-induced neurotoxicity by restoring *PINK1/Parkin* signaling, thereby enhancing mitophagy and suppressing apoptosis. The present study elucidates the central mechanism of SIM-induced PD-like neurotoxicity in vitro and, for the first time, confirms the potential protective effect of SAL. These findings provide a novel theoretical basis for investigating nerve injury induced by SIM exposure and underscore the potential of plant-derived compounds in preventing nerve injuries related to environmental toxicants.

## 1. Introduction

Parkinson’s disease (PD) is a prevalent neurodegenerative disorder and has emerged as a significant public health concern [1]. In addition to genetic predispositions, exposure to pesticides and environmental toxins constitutes a critical risk factor for the onset and progression of PD [2,3,4]. Among these, triazine herbicides have garnered attention due to their persistence and heightened toxicity [5].

Simazine (SIM), chemically known as 2-chloro-4,6-di(ethylamino)-1,3,5-triazine, is a selective systemic herbicide [6]. Both epidemiological and experimental studies have demonstrated a significant correlation between simazine exposure and the risk of developing PD [7], thereby necessitating further exploration of the neurotoxic effects of SIM and its underlying toxicological mechanisms [8,9].

The core pathological feature of PD is the selective loss of dopaminergic neurons in the substantia nigra, with apoptosis representing a significant mechanism of neuronal death in this context [10,11]. An imbalance in mitophagy homeostasis plays a crucial role in dopaminergic neuronal degeneration [12]: when mitochondrial function and structure become impaired, the inability to timely clear damaged mitochondria can trigger a cascade of oxidative stress within dopaminergic neurons, ultimately leading to apoptosis. Existing studies on mitophagy highlight the *PINK1/Parkin* pathway as a key mechanism for maintaining mitochondrial homeostasis and facilitating the clearance of damaged mitochondria [13]. In light of this, the present study will focus on the mechanisms of mitophagy and investigate whether SIM induces cellular injury in dopaminergic neuron-like SH-SY5Y cells by disrupting this critical physiological process.

Furthermore, phytochemicals have been recognized to play a role in offering protection against exposure to environmental toxicants and hold potential as natural preventive agents [8]. Salidroside (SAL), an active ingredient from Rhodiola plants, has been proven through several scientific studies to possess antioxidant, anti-aging, anti-tumor, and anti-apoptotic properties [14,15]. Some studies suggest that SAL may have neuroprotective effects [16]. Not only does it promote the transition of stem cells into dopaminergic neurons along with increasing the number of neurons in the substantia nigra, but it also prevents the apoptosis of neurons and is believed to work by activating the DJ-1-Nrf2 pathway, thus preventing oxidative stress. In addition, SAL regulates iron metabolism, inhibits ferroptosis, and diminishes neuroinflammation in models of TBI [17,18]. SAL exhibits multi-target neuroprotective effects. Accordingly, we further expect SAL to have a protective effect on SIM-induced cellular injury in dopaminergic neuron-like cells.

SH-SY5Y cells, a subclonal cell line derived from human neuroblastoma, exhibit typical neuron-like morphology and electrophysiological characteristics. They possess a consistent genetic background and can be cultured in a standardized manner, making them suitable for studying the biological characteristics of these cells. More importantly, they can stably express dopaminergic neuron-specific markers, such as tyrosine 3-monooxygenase and dopamine transporter (SLC6A3), which positions them as an ideal model for simulating the core pathology of dopaminergic neuron injury in Parkinson’s disease [8]. In this study, we employed bioinformatics analysis to elucidate the underlying molecular mechanisms, utilizing SH-SY5Y cells as an in vitro neuron-like model to investigate the direct effects of SIM. A thorough analysis of damage, coupled with molecular biology experiments and the incorporation of IBR analysis to comprehensively quantify the overall cellular damage, is crucial for assessing the complex mechanisms of toxicity at the cellular level. This approach enhances our understanding of the damage mechanisms involved. Consequently, we preliminarily explored the preventive effect of SAL on the imbalance of mitophagy and apoptosis in dopaminergic neuron-like SH-SY5Y cells induced by SIM, along with the associated mechanisms [15]. Our findings offer a novel perspective and theoretical foundation for the development of natural low-toxicity drugs aimed at providing in vitro mechanistic evidence for understanding SIM-induced neurotoxicity and the protective effects of SAL [19].

## 2. Results

### 2.1. Network Toxicology Analysis of the Potential Mechanisms by Which SIM Induces PD

**Figure 1:**
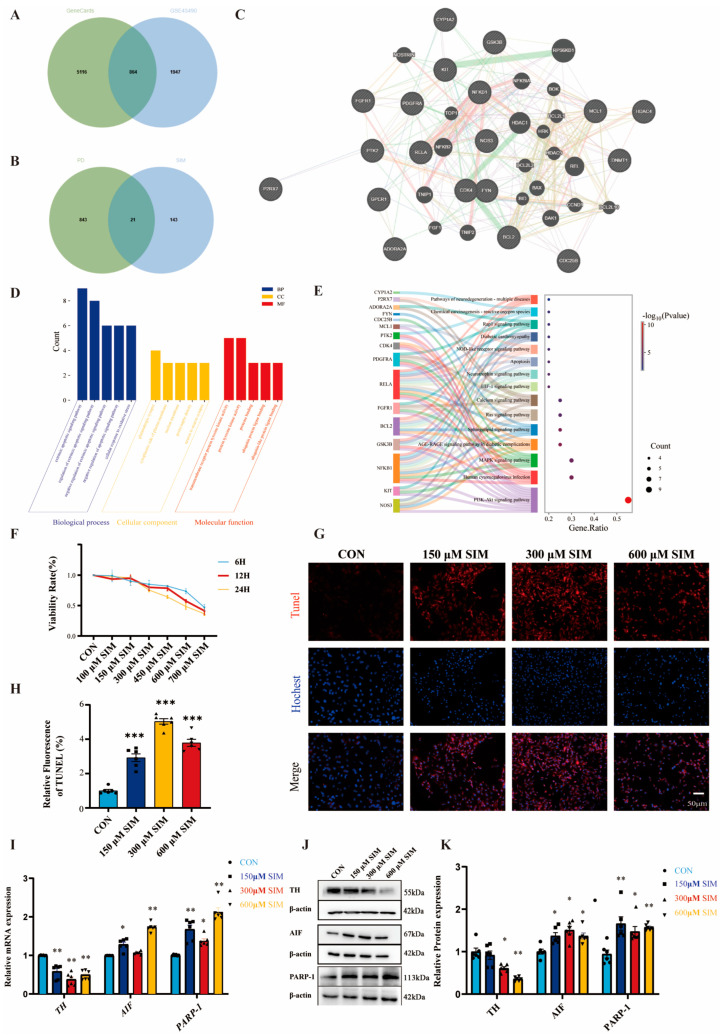
**SIM network toxicology analysis results and SIM-induced apoptosis.** (**A**) Venn diagram for screening PD-related genes. (**B**) Venn diagram for screening SIM-induced PD-related genes. (**C**) GeneMANIA network diagram of SIM-induced PD-related genes. Color-coded interaction evidence types, including Physical Interactions (pink), Genetic Interactions (green), Co-expression (light purple), Shared protein domains (beige), Pathway (light blue), Predicted (orange), and Co-localization (blue). (**D**) GO enrichment circle diagram of SIM-induced related genes. (**E**) KEGG enrichment results of SIM-induced related genes. (**F**) The cell viability of SH-SY5Y was detected by CCK-8 assay. (**G**,**H**) Apoptosis of cells is detected by TUNEL. (**I**,**J**,**K**) qRT-PCR detection of *TH*, *AIF*, *PARP-1* mRNA and WB analysis of TH, AIF, PARP-1. Bars indicate mean ± SEM. * statistically significant difference compared with the control, * *p* < 0.05, ** *p* < 0.01, *** *p* < 0.001, n = 6.

This study analyzed sequencing data from PD patients in the GEO database (GSE43490), identifying 2813 differentially expressed genes. These were intersected with PD-related targets from GeneCards to increase confidence, resulting in 865 high-confidence PD targets (Figure 1A). Subsequently, potential targets of SIM were collected by integrating the Comparative Toxicogenomics Database (CTD), SwissTargetPrediction, and TargetNet, yielding 164 SIM-related targets. The intersection of these two datasets revealed 21 overlapping targets (Figure 1B). A co-expression network of these shared targets was constructed using GeneMANIA (Figure 1C). In this network, physical interactions were predominant (27.44%), followed by genetic interactions (22.20%) and co-expression (18.34%). Additional associations included shared protein domains (14.19%), pathway co-involvement (9.57%), predicted interactions (4.98%), and co-localization (3.28%), highlighting a complex and multifaceted regulatory architecture among the overlapping genes. Core co-expressed genes (e.g., RPS6KB1, HDAC1, REL, NFKB2) participate in key PD-related pathways, including inflammation regulation (NFKB1, RELA), apoptosis (BCL2, BAX, BAK1), and signal transduction (FGFR1, GSK3B), suggesting their central coordinating role in SIM-induced PD-like neurotoxicity.

Further analysis of the overlapping targets showed that Gene Ontology (GO) enrichment in biological processes (BP) was primarily associated with regulation of the extrinsic apoptotic signaling pathway, response to oxidative stress, and glutamatergic synapse-related processes, all of which are critical for dopaminergic neuron survival and basal ganglia circuit homeostasis in PD. At the cellular component (CC) level, the genes were localized to the cytoplasmic side of the plasma membrane, nuclear envelope, postsynaptic density, and neuronal synapses, providing structural support for synaptic transmission and mitochondrial functional stability. Molecular function (MF) terms were enriched in tyrosine kinase activity and ubiquitin-binding functions, which mediate protein phosphorylation cascades and regulate the stability of autophagy-related proteins, thereby contributing to mitochondrial quality control (Figure 1D). KEGG pathway enrichment confirmed significant involvement in the PI3K-Akt, MAPK, AGE-RAGE, calcium signaling, and neurodegenerative disease pathways (Figure 1E). Dysregulation of PI3K-Akt and MAPK pathways alters phosphorylation of mitophagy-related proteins, while the AGE-RAGE pathway exacerbates oxidative stress and impairs mitochondrial function in dopaminergic neuron-like cells—both aligning with core PD pathological features. In summary, SIM may disrupt the above signaling network, impair mitophagy homeostasis in dopaminergic neuron-like SH-SY5Y cells, trigger synaptic dysfunction and neuronal apoptosis, and thereby contribute to PD-like neurotoxicity.

### 2.2. SIM Induces Apoptosis in SH-SY5Y Cells

To investigate and verify the mechanisms by which SIM mediates PD development, we established an in vitro SH-SY5Y cell model. CCK-8 assay, TUNEL staining, qRT-PCR, and Western blotting were used to evaluate the effects of SIM on SH-SY5Y cells and confirm its ability to induce apoptosis. CCK-8 results showed that SH-SY5Y cell viability significantly decreased in a dose- and time-dependent manner upon SIM exposure (Figure 1F). Specifically, after treatment with 600 μM SIM for 24 h, cell viability dropped significantly to approximately 50% (48.60% ± 7.79%). Moreover, TUNEL staining was used to assess apoptosis, revealing a significant increase in apoptotic rates across SIM-treated groups (Figure 1G,H). Concurrently, qRT-PCR and Western blot results demonstrated that mRNA and protein levels of key markers related to dopaminergic neurons and apoptosis were significantly altered under different SIM doses, further confirming that SIM induces apoptosis in SH-SY5Y cells (Figure 1I–K).

### 2.3. SIM Damages Mitochondrial Structure and Causes Mitochondrial Metabolic Imbalance in SH-SY5Y Cells

**Figure 2:**
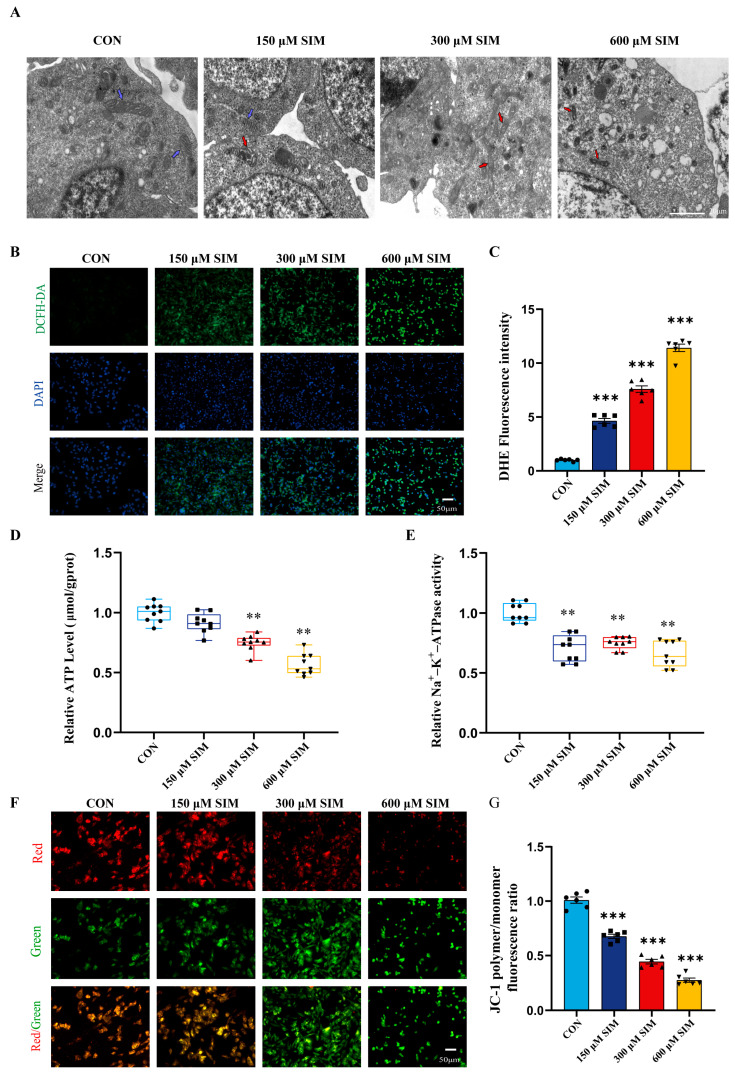
**SIM damaged the mitochondrial structure and caused the mitochondrial metabolic imbalance of SH-SY5Y cells.** (**A**) Observation of the damage to mitochondrial structure induced by SIM under an electron microscope. Blue arrows with black edges: normal mitochondria; Red arrows with black edges: swelling and vacuole. (**B**,**C**) The results of ROS detection. (**D**) The results of intracellular ATP detection. (**E**) The results of the activity of Na^+^-K^+^-ATPase detection. (**F**,**G**) The analysis of MMP detection. Bars indicated mean ± SEM. * statistically significant difference compared with the control, ** *p* < 0.01, *** *p* < 0.001, n = 6.

Results showed that mitochondria in SIM-exposed SH-SY5Y cells exhibited significant morphological changes, including swelling and vacuolization, indicating structural damage (Figure 2A). Additionally, intracellular ROS levels were significantly elevated in a dose-dependent manner, further suggesting mitochondrial functional impairment (Figure 2B,C). We also measured changes in ATP levels, Na^+^-K^+^-ATPase activity, and mitochondrial membrane potential. All these parameters showed significant dose-dependent decreases under SIM treatment, directly reflecting disruption of mitochondrial metabolic homeostasis (Figure 2D–G). These findings indicate that SIM causes multidimensional mitochondrial damage, including structural injury, oxidative stress, energy metabolism disorders, and impaired ion pump function—which collectively exacerbate cellular injury.

### 2.4. SIM Inhibited the Mitophagy of SH-SY5Y Cells via PINK1

**Figure 3:**
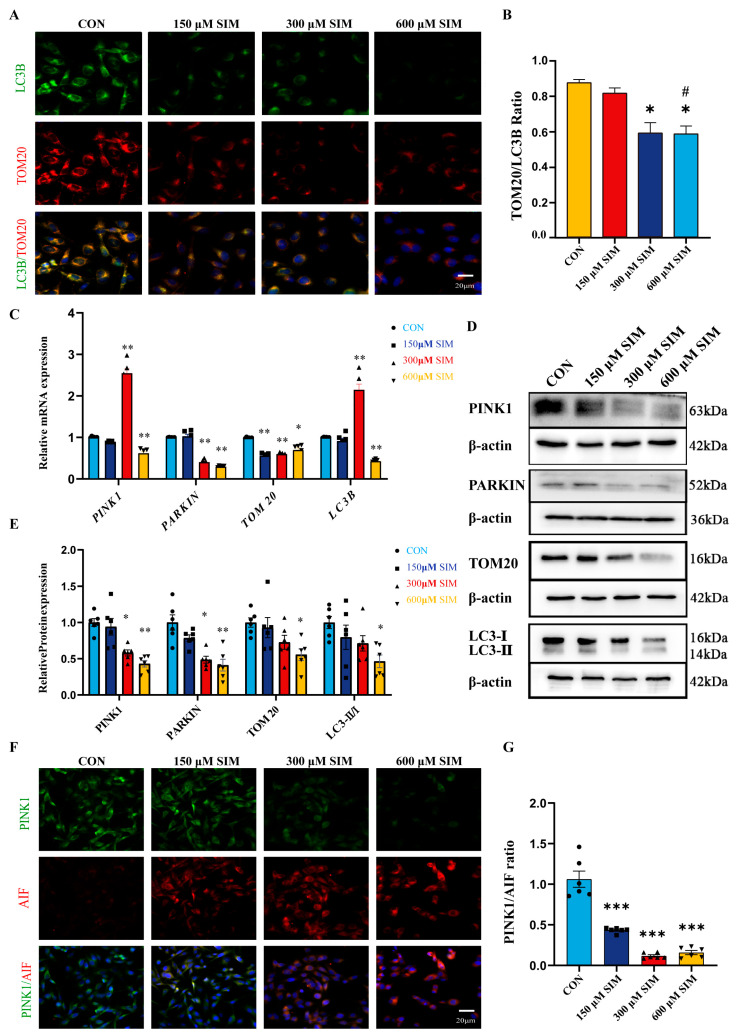
**SIM inhibited the mitophagy of SH-SY5Y cells.** (**A**,**B**) IF detected the co-expression of LC3B and TOM20. (**C**) qRT-PCR detected the mRNA levels of *PINK1*, *Parkin*, *TOM20* and *LC3B* related to mitophagy. (**D**,**E**) WB detected the protein expression levels of PINK1, Parkin, TOM20 and LC3B-II/I related to mitophagy. (**F**,**G**) IF detected the co-expression of PINK1 and AIF. Bars indicated mean ± SEM. # statistically significant difference compared with the SIM group, # *p* < 0.05; * statistically significant difference compared with the control, * *p* < 0.05, ** *p* < 0.01, *** *p* < 0.001, n = 6.

Mitochondrial structural and functional damage may be accompanied by disruption of mitochondrial homeostasis or mitophagy. Based on network toxicology predictions, mitophagy may be a key pathway through which SIM induces SH-SY5Y cell apoptosis. To test this hypothesis, we designed follow-up experiments. Although SIM caused multidimensional mitochondrial damage, its direct effect on mitophagy remained unconfirmed. We therefore evaluated the expression of mitophagy-related proteins and activity of associated pathways to determine whether SIM exerts neurotoxicity by interfering with mitophagy. Dual immunofluorescence staining for LC3B/TOM20 revealed that SIM suppressed mitophagy in SH-SY5Y cells (Figure 3A,B). qRT-PCR and Western blot results further confirmed that SIM significantly downregulated the key mitophagy markers *LC3B-II/I* and *TOM20* and attenuated *PINK1/Parkin* pathway activity (Figure 3C–E). These findings indicate that SIM negatively regulates mitophagy. To validate this, we performed additional immunofluorescence analysis. Compared to controls, PINK1 expression was significantly reduced while AIF expression was significantly increased in the SIM-exposed group, further supporting the conclusion that SIM inhibits mitophagy in SH-SY5Y cells (Figure 3F,G).

### 2.5. Network Pharmacology Analysis of the Potential Protective Mechanism of SAL in PD

**Figure 4:**
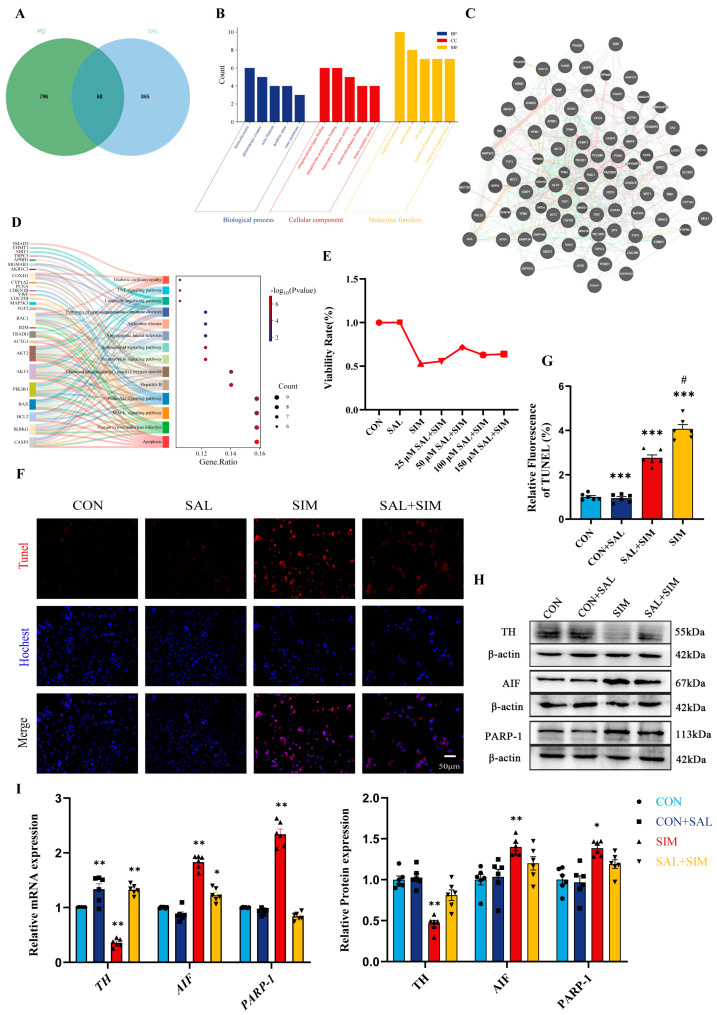
**SAL network toxicology analysis results and SAL-induced protective effects in PD.** (**A**) Venn diagram for screening SAL-protected PD-related genes. (**B**) GeneMANIA network diagram of SAL-protected PD-related genes. (**C**) GO enrichment circle diagram of SAL-protected PD-related genes. Color-coded interaction evidence types, including Co-expression (light purple), Physical Interactions (pink), Predicted (orange), Genetic Interactions (green), Co-localization (blue), Pathway (light blue), and Shared protein domains (beige). (**D**) KEGG enrichment results of SAL-protected PD-related genes. (**E**) The cells viability of SH-SY5Y was detected by CCK-8 assay. (**F**,**G**) Apoptosis of cells is detected by TUNEL. (**H**,**I**) qRT-PCR detection of *TH*, *AIF*, *PARP-1* mRNA and WB analysis of TH, AIF, PARP-1. Bars indicated mean ± SEM. # statistically significant difference compared with the SIM group, # *p* < 0.05; * statistically significant difference compared with the control, * *p* < 0.05, ** *p* < 0.01, *** *p* < 0.001; n = 6.

Based on our network toxicology analysis and in vitro experiments, we found that SIM induces neurotoxicity and triggers apoptosis in SH-SY5Y cells by suppressing mitophagy. A literature review revealed that SAL, a natural compound with protective properties, exerts biological effects that closely overlap with—but directly oppose—SIM’s toxic mechanisms. To systematically elucidate the molecular mechanisms by which SAL intervenes in SIM-induced PD-like neurotoxicity in SH-SY5Y cells, we employed a network pharmacology approach to screen SAL’s key targets and perform enrichment analyses to dissect its biological properties and pathway regulation patterns. By combining SwissTargetPrediction and TargetNet, we identified 933 potential SAL targets. Intersecting these with PD-related targets yielded 69 overlapping genes (Figure 4A). A co-expression network of these core PD-protective targets was constructed using GeneMANIA (Figure 4C). Co-expression dominated the network (37.87%), followed by physical interactions (36.60%), along with predicted interactions (9.82%), genetic interactions (7.75%), co-localization (4.07%), pathway associations (2.35%), and shared protein domains (1.53%), highlighting a complex regulatory network among these genes. Core co-expressed genes (e.g., SFPQ, PACSIN1, CLK3, BID) participate in key PD-related pathways, including apoptosis regulation (BAX, BCL2, CASP3) and neuroprotection (FGF2, SIGMAR1, ADORA2A), suggesting their central coordinating role in SAL-mediated protection against PD-like cellular injury.

Further analysis of the overlapping targets showed that GO enrichment in BP was mainly associated with regulation of neuronal death and response to oxidative stress—processes directly involved in SIM-induced apoptosis in dopaminergic neuron-like SH-SY5Y cells, providing a biological basis for SAL’s antagonism of SIM toxicity and its regulation of mitophagy and apoptosis. At the CC level, genes were localized to the outer membrane and dendritic spines, offering molecular support for SAL’s role in maintaining mitochondrial membrane stability and neuronal structural integrity against SIM-induced apoptotic damage. MF terms were enriched in ubiquitin binding and kinase regulatory activity, mediating protein modification and phosphorylation cascades that contribute to SAL’s homeostatic regulation of mitophagy and apoptosis signaling (Figure 4B). KEGG analysis confirmed enrichment in the PI3K-Akt, MAPK, AGE-RAGE, and apoptosis pathways (Figure 4D). Aberrant PI3K-Akt and MAPK signaling are central to SIM-induced mitophagy dysregulation and neuronal apoptosis; SAL may counteract SIM toxicity by modulating these pathways and suppressing oxidative stress-mediated mitochondrial damage. In summary, SAL likely exerts protective effects against SIM-induced PD-like neurotoxicity by regulating core signaling pathways, restoring mitophagy homeostasis in dopaminergic neuron-like SH-SY5Y cells impaired by SIM, and inhibiting apoptosis.

### 2.6. SAL Reduces SIM-Induced Mitochondrial Structural Damage and Cell Apoptosis

CCK-8 assays showed that pre-treatment with different concentrations of SAL (25, 50, 100, and 150 μM) alleviated SIM-induced cytotoxicity (Figure 4E). Notably, 50 μM SAL demonstrated the most significant protective effect in enhancing cell viability and preventing apoptosis. Thus, this concentration was selected as the optimal protective dose for subsequent experiments. At the molecular level, SAL pre-treatment not only reversed the SIM-induced decrease in TH expression but also reduced the expression of AIF and PARP-1. These results, together with TUNEL findings, confirm that SAL pretreatment ameliorates SIM-induced apoptosis in SH-SY5Y cells (Figure 4F–I).

### 2.7. SAL Prevents SIM-Induced Mitochondrial Metabolic Imbalance

**Figure 5:**
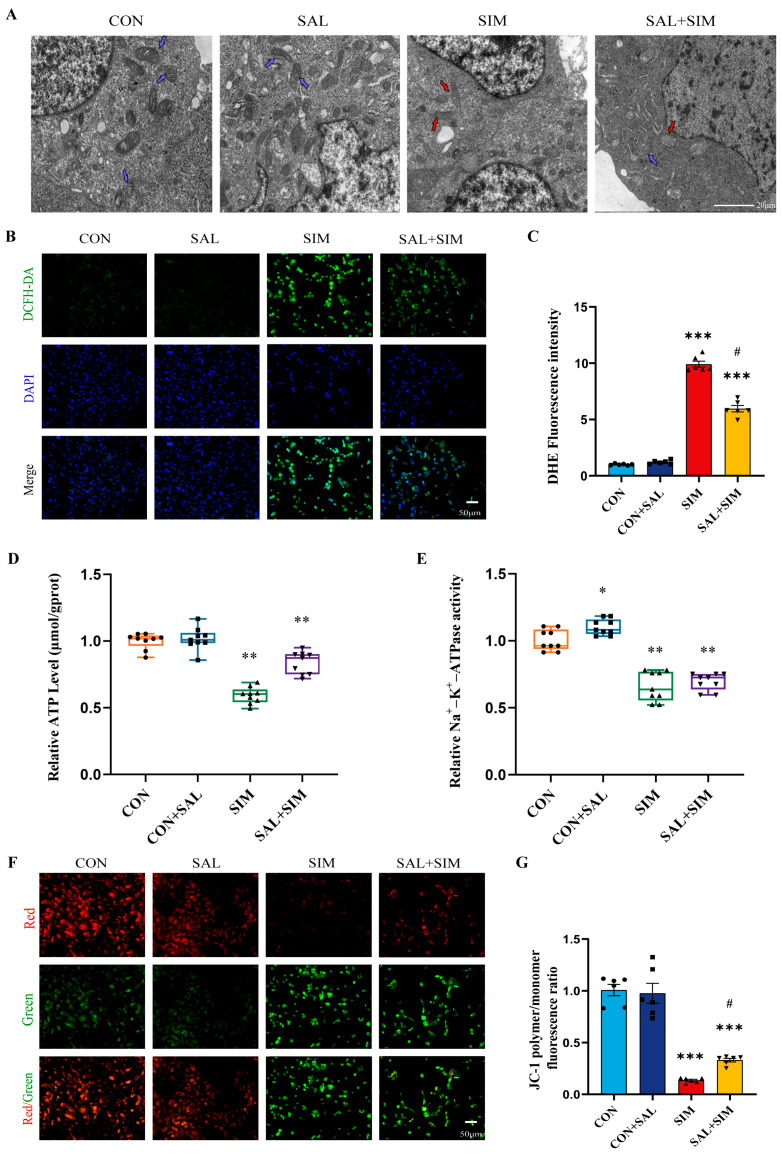
**SAL prevented the mitochondrial metabolic imbalance induced by SIM.** (**A**) Observation of the damage to mitochondrial structure induced by SIM under an electron microscope. Blue arrows with black edges: normal mitochondria; Red arrows with black edges: swelling and vacuole. (**B**,**C**) The results of ROS detection. (**D**) The results of intracellular ATP detection. (**E**) The results of the activity of Na^+^-K^+^-ATPase detection. (**F**,**G**) The analysis of MMP detection. Bars indicated mean ± SEM. # statistically significant difference compared with the SIM group, # *p* < 0.05; * statistically significant difference compared with the control, * *p* < 0.05, ** *p* < 0.01, *** *p* < 0.001, n = 4.

Compared to the mitochondrial damage caused by SIM, SAL pre-treatment significantly mitigated SIM-induced mitochondrial structural damage in SH-SY5Y cells (Figure 5A). SAL pre-treatment effectively counteracted SIM-induced mitochondrial metabolic imbalance and significantly reduced the SIM-induced elevation in ROS levels, indicating a strong protective role against mitochondrial injury (Figure 5B,C). Furthermore, measurements of cellular ATP levels, Na^+^-K^+^-ATPase activity, and mitochondrial membrane potential showed that SAL pre-treatment partially maintained mitochondrial metabolic balance and preserved mitochondrial transmembrane potential, ensuring mitochondrial membrane integrity and normal function (Figure 5D–G). This reveals SAL’s protective potential against SH-SY5Y cell mitochondrial dysfunction. These results demonstrate SAL’s protective effects against SIM-induced neurotoxicity in SH-SY5Y cells from both functional and structural perspectives.

### 2.8. SAL Prevents SIM-Induced Suppression of Mitophagy

**Figure 6:**
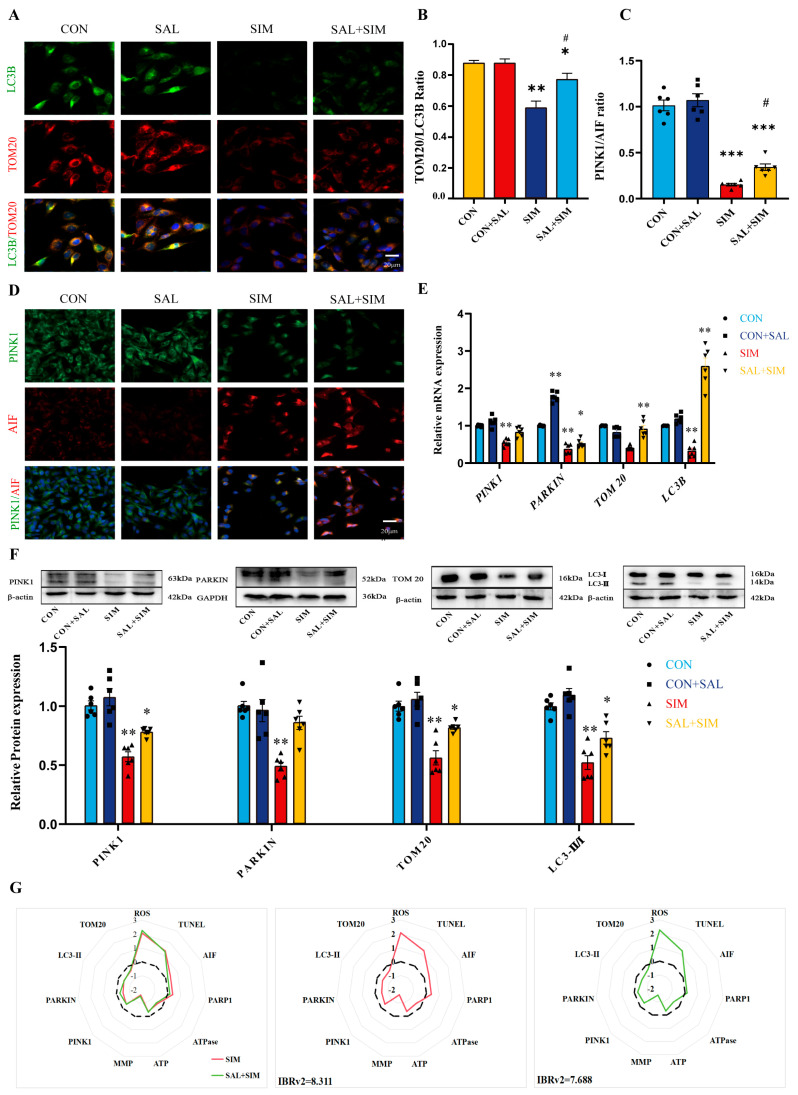
**SAL prevented SIM-induced inhibition of mitophagy.** (**A**,**B**) IF detected the co-expression of LC3B and TOM20. (**C**,**D**) IF detected the co-expression of PINK1 and AIF. (**E**,**F**) qRT-PCR detected the mRNA levels of *PINK1*, *Parkin*, *TOM20* and *LC3B* related to mitophagy, and WB detected the protein expression levels of PINK1, Parkin, TOM20 and LC3B-II/I related to mitophagy. (**G**) IBR analyzed the preventive effect of SAL on the comprehensive effects of SIM on SH-SY5Y cells. Bars indicate mean ± SEM. The black dashed line represents the 0-axis reference line, serving as the baseline for normalized values. # statistically significant difference compared with the SIM group, # *p* < 0.05; * statistically significant difference compared with the control, * *p* < 0.05, ** *p* < 0.01, *** *p* < 0.001, n = 4.

Based on immunofluorescence co-localization of LC3B/TOM20 and PINK1/AIF, SAL pre-treatment alleviated the SIM-induced inhibition of mitophagy in SH-SY5Y cells and reduced expression of the apoptotic marker AIF (Figure 6A–D). This suggests that SAL reduces SIM-induced apoptosis by modulating mitophagy. qRT-PCR and Western blot results further confirmed this conclusion (Figure 6E,F). We also integrated multidimensional indicators from the SIM-only and SAL+SIM groups—including mitochondrial apoptosis, mitochondrial metabolism, mitophagy, and general apoptosis—for comprehensive biomarker analysis to evaluate the impact of SAL pre-treatment. The IBR score was 8.311 in the SIM group and 7.688 in the SAL+SIM group (higher IBR indicates greater toxicity) (Figure 6G). Radar plot analysis provided a comprehensive and intuitive visualization of SAL’s protective effects against SIM-induced mitochondrial dysfunction and apoptosis.

### 2.9. SAL Blocks SIM-Induced Suppression of Mitophagy and Apoptosis via the PINK1/Parkin Pathway

**Figure 7:**
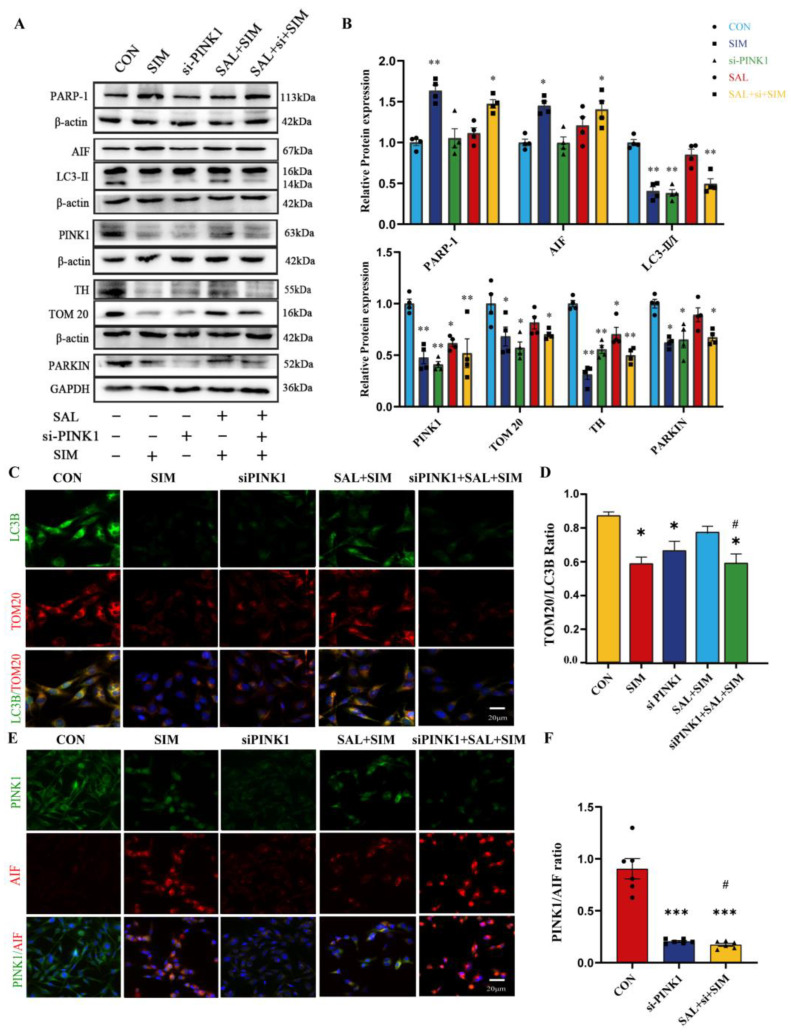
**SAL prevented SIM-induced inhibition of mitophagy and apoptosis via the *PINK1/Parkin* pathway.** (**A**,**B**) WB detected the expressions of proteins related to mitophagy and apoptosis. (**C**,**D**) IF detected the co-expression of LC3B and TOM20. (**E**,**F**) IF detected the co-expression of PINK1 and AIF. Bars indicate mean ± SEM. # statistically significant difference compared with the SAL+SIM group, # *p* < 0.05; * statistically significant difference compared with the control, * *p* < 0.05, ** *p* < 0.01, *** *p* < 0.001, n = 4.

To validate the above findings, we used molecular docking to evaluate the binding between SIM, SAL and the corresponding target protein. The binding energy was less than −5, and the SAL score was higher than that of SIM, indicating good binding (Figure S1). We used siRNA to knock down *PINK1* expression. After transfection, this study verified its transfection efficiency in SH-SY5Y cells by immunofluorescence and qRT-PCR (Figure S2). After the successful construction of PINK1-knockdown cell model, follow-up experiments were carried out. *siPINK1* intervention significantly reduced mRNA and protein levels of the autophagy proteins Parkin, LC3B, and TOM20, and abolished SAL’s ability to reverse the SIM-induced downregulation of Parkin, LC3B, and TOM20, as well as the reduction in apoptotic proteins AIF and PARP-1 (Figure 7A,B). Consistently, dual immunofluorescence staining for LC3B/TOM20 and PINK1/AIF yielded the same results. Collectively, multiple lines of evidence demonstrate that SAL alleviates SIM-induced mitophagy suppression and apoptosis in SH-SY5Y cells by modulating the *PINK1/Parkin* signaling pathway (Figure 7C–F).

## 3. Discussion

This study integrated bioinformatics analysis and in vitro experimental validation to confirm that SIM induces mitochondrial dysfunction in SH-SY5Y cells and ultimately triggers apoptosis by inhibiting the *PINK1/Parkin*-mediated mitophagy pathway. In contrast, SAL, a natural plant bioactive component, exhibits a significant preventive effect and effectively blocks this cascade of injuries induced by SIM. These findings reveal the underlying mechanisms associated with SIM-induced neurotoxicity and identify SAL as a promising natural compound suitable for potential protective intervention in vitro.

SIM has been recognized as an important environmental risk factor for PD [20,21,22]. The specific mechanisms underlying SIM-induced neurotoxicity remain unclear, and natural compounds suitable for potential protective intervention in vitro have not been identified [7,23].

In previous studies, SIM has been identified as a significant environmental risk factor for PD, capable of inducing damage to dopaminergic neurons [24]. However, the specific mechanisms underlying SIM-induced neurotoxicity remain unclear. Network toxicology offers a more comprehensive perspective for assessing disease risk and elucidating the molecular pathways associated with chemical toxicants. Notably, the genes targeted by SIM exhibit substantial overlap with PD-related genes. Additionally, results from co-expression network analysis and pathway enrichment analysis indicate the potential occurrence of cellular injury and apoptosis in dopaminergic neuron-like cells. Mitochondrial dysfunction is often accompanied by an imbalance in mitophagy homeostasis, and mitophagy inhibition is one of the important regulatory mechanisms mediating apoptosis [25,26]. Previous studies have demonstrated that dysregulated mitophagy and apoptosis in dopaminergic neurons of the substantia nigra play a critical role in PD pathogenesis [27]. The regulation of mitophagy is highly complex and can be disrupted by various external stressors. Mitochondrial dysfunction may impair mitophagy, while defective mitophagy further exacerbates mitochondrial damage. This vicious cycle ultimately promotes neuronal apoptosis and contributes to neurodegeneration [28,29]. When we carried out an enrichment analysis, we discovered that the specific term ‘mitophagy’ was not necessarily highlighted to begin with. However, upon deeper scrutiny of the results, we found that the functions and pathways involved are, in fact, intimately connected to mitophagy in the pathophysiological context.

Among the existing studies on the governing mitophagy of mitophagy, the *PINK1/Parkin* pathway is one of the most extensively studied mechanisms regulating mitophagy [30,31]. The *PINK1/Parkin* pathway serves as a key mechanism for maintaining mitochondrial homeostasis and plays a crucial role in scavenging damaged mitochondria [28,32,33], enabling the selective recognition and clearance of damaged mitochondria, as well as the regulation of mitochondrial homeostasis, thus inhibiting the abnormal release of pro-apoptotic factors such as cytochrome c and AIF. Moreover, previous studies have shown that the expression of PINK1 and Parkin is significantly downregulated in the MPP^+^ MN9D cell PD study model in vitro. Mitochondrial dysfunction and respiratory chain damage caused by PINK1 or Parkin inactivation can activate a mitochondria-nuclear signal transduction cascade that ultimately leads to aberrant activation of PARP-1 apoptosis signaling [34]. Furthermore, abnormal expressions of the *PINK1/Parkin* pathway and mitochondrial stress disorders have been observed found in both in vitro and in vivo PD study models as well as in postmortem brain tissues of PD patients [24]. In further vitro experiments, we found that SIM could significantly inhibit the *PINK1/Parkin* pathway. In addition, SIM altered the expression of LC3B and TOM20, indicating impaired mitophagy and mitochondrial stress responses, accompanied by disrupting the normal process, increased ROS, and enhanced apoptotic signaling. Combined with network toxicology results and enrichment analysis, it was proved that SIM could exacerbate oxidative stress by interfering with mitochondrial energy metabolism, thereby disrupting the normal process of mitophagy. Ultimately, this leads to apoptosis of dopaminergic neurons and induces PD-like damage.

Given the central regulatory role of the *PINK1/Parkin* pathway in PD pathogenesis, and the finding in this study that SIM can induce PD-like lesions by inhibiting this pathway, previous studies have confirmed that the regulatory effects of different substances on this pathway may show opposite trends and produce different pathological or protective effects, suggesting that potential protective substances capable of preventing SIM-induced injury could be further explored [13,30,31]. In the MPTP-induced PD animal model, SAL could significantly promote the translocation of Parkin to mitochondria, upregulate the number of TH^+^ neurons, and restore the balance of mitophagy flux and energy metabolism, ultimately inhibit the degeneration of dopaminergic neurons in the substantia nigra and striatum [9]. Based on this, we further speculated whether Sal could prevent dopaminergic neurotoxicity by interfering with SIM-induced inhibition of mitophagy. Coincidentally, the experimental results of our study are consistent with this hypothesis. First, in the network pharmacology of SAL to prevent PD, we found a high degree of overlap between its targets and related pathways and those of SIM network toxicology, suggesting that SAL may play an important role in PD prevention. Molecular docking analysis showed that SAL exhibited strong binding affinity for both PINK1 and Parkin, and the combination of SAL and SIM showed a higher affinity for both PINK1 and Parkin proteins. Furthermore, experiments showed that pretreatment with SAL significantly alleviated SIM-induced mitochondrial structural damage. It also alleviated metabolic and autophagic imbalances and reduced apoptosis in SH-SY5Y cells. To further test this hypothesis, we found that SAL had lost of preventive effect on SIM-induced mitochondrial structural damage, metabolic, and autophagic imbalances by silencing *PINK1* expression with targeted siRNA, suggesting that SAL may play an important role in regulating mitochondrial structural damage, These findings suggest that the protective effect of SAL against SIM-induced dopaminergic neuronal toxicity is partly related to its action and that of the PINK1 target.

However, this study still has some limitations. On the one hand, this study only explored the commonly used in vitro cell models and PINK1-related regulatory mechanisms, in vivo animal experiments have not been carried out to further verify the protective effect of SAL and the specific mechanism. On the other hand, whether Sal achieves the prevention of SIM-induced injury by regulating the activity of mRNA transcription promoter and other mechanisms, still needs to be further studied. The above shortcomings will be gradually improved in the follow-up research work and further expand the relevant mechanism research.

## 4. Materials and Methods

### 4.1. Network Toxicology and Network Pharmacology Analyses

#### 4.1.1. Network Toxicology Analysis

To investigate the potential neurotoxic mechanisms of SIM in PD, we predicted target genes of SIM by integrating three complementary resources: the Comparative Toxicogenomics Database (CTD, https://ctdbase.org/), SwissTargetPrediction (http://www.swisstargetprediction.ch), and TargetNet (http://targetnet.scbdd.com/). The databases were accessed on 14 December 2025, with “simazine” used as the search keyword.

Separately, we obtained gene expression microarray data from PD patients (GEO dataset GSE43490) and performed differential expression analysis to identify differentially expressed genes (DEGs). These DEGs were intersected with PD-associated genes curated from the GeneCards database to define a high-confidence PD-related gene set.

Functional associations and protein–protein interactions among these overlapping genes were further explored using GeneMANIA (https://genemania.org/ accessed on 20 December 2025) [35]. The intersection between the SIM-predicted target genes and the high-confidence PD gene set yielded candidate targets potentially mediating SIM-induced PD pathogenesis. This core gene set was subjected to functional enrichment analysis using Metascape (http://metascape.org/gp/index.html, 20 December 2025), including Gene Ontology (GO) annotation and KEGG pathway enrichment.

#### 4.1.2. Network Pharmacology Analysis

For SAL, potential therapeutic targets were jointly predicted using SwissTargetPrediction and TargetNet. Redundant entries were removed to generate a non-redundant SAL target gene set. This set was then intersected with the aforementioned high-confidence PD-related gene set to identify candidate therapeutic targets of SAL in PD.

GeneMANIA was employed to assess functional linkages among the core targets. Functional enrichment of these overlapping targets was performed using Metascape for GO and KEGG pathway analyses. In addition, molecular docking and visualization were performed using the AutoDock vina (4.6.0) and PyMol (3.0.5) for further screening.

### 4.2. Cell Culture and Treatment

The human neuroblastoma cell line SH-SY5Y was obtained from the China Center for Type Culture Collection (CCTCC). Cells were maintained in MEM/F12 medium (Lot No.: PM151220) supplemented with 10% fetal bovine serum (FBS), 100 IU/mL penicillin, and 100 μg/mL streptomycin (CAS: C0222; Beyotime, Shanghai, China) at 37 °C in a humidified atmosphere containing 5% CO_2_.

For experimental treatments, cells were exposed to simazine (SIM; purity ≥ 98%) at concentrations of 150, 300, and 600 μM for 24 h. For rescue experiments, cells were pretreated with SAL for 24 h, then exposed to 600 μM SIM for an additional 24 h. The SAL concentration used was determined based on prior literature and preliminary CCK-8 assays; 50 μM SAL demonstrated optimal protective effects against 600 μM SIM-induced cytotoxicity and was therefore selected for all subsequent experiments.

### 4.3. Cell Transfection

SH-SY5Y cells were seeded into 6-well plates at a density of 5 × 10^5^ cells per well and cultured in MEM/F12 medium containing 10% FBS at 37 °C under 5% CO_2_. Upon reaching 70–80% confluence, cells were transfected with 50 nM small interfering RNA (siRNA; CAS: R10043.10; targeting PINK1) using FECT™ CP transfection reagent (RiboBio, Guangzhou, China), according to the manufacturer’s protocol. After transfection, cells were incubated for the indicated time before proceeding to downstream assays.

### 4.4. Cell Viability Assay

Following experimental treatments, cell viability was assessed using a CCK-8 kit (CAS: BS350B; Biosharp, Shanghai, China) according to the manufacturer’s instructions.

### 4.5. Detection of Mitochondrial Membrane Potential (ΔΨm)

Mitochondrial membrane potential (MMP) was evaluated using a JC-1 assay kit (CAS: C2003S; Beyotime, Shanghai, China). The ratio of red fluorescence (JC-1 aggregates, indicating high ΔΨm) to green fluorescence (JC-1 monomers, indicating depolarized mitochondria) was used to quantify the extent of mitochondrial depolarization.

### 4.6. TUNEL Assay

Cells were fixed with 4% paraformaldehyde (CAS: G1101; Servicebio, Wuhan, China) for 15 min at room temperature, followed by three 5 min washes with PBS. Permeabilization was performed with 0.1% Triton X-100 (CAS: P0096; Beyotime, Shanghai, China) for 15 min at 37 °C, followed by another three 5 min PBS washes. Cells were then incubated with 200 μL of TUNEL reaction mixture (CAS: E-CK-A322-100; Elabscience, Wuhan, China) in a humidified chamber at 37 °C in the dark for 2 h. Nuclei were counterstained with Hoechst 33342 (CAS: C1022; Beyotime, Shanghai, China). After three 3 min PBS washes, apoptotic cells were visualized and imaged using a fluorescence microscope.

### 4.7. Quantitative Real-Time PCR (qRT-PCR)

Total RNA was extracted from cells and reverse-transcribed into cDNA using the PrimeScript RT Reagent Kit with gDNA Eraser (Takara Bio, Otsu, Japan). Primer sequences are listed in Table 1. qPCR was performed on an ABI 7500 Real-Time PCR System (Thermo Fisher Scientific, Waltham, MA, USA) under standard cycling conditions. Gene expression levels were normalized to β-actin and calculated using the 2^−ΔΔCT^ method.

### 4.8. Western Blot

Cells were lysed in RIPA buffer containing 1% phenylmethanesulfonyl fluoride (PMSF). Equal amounts of protein were separated by 8% SDS-PAGE at 160 V for 30–40 min and then electrotransferred onto PVDF membranes (Millipore, Billerica, MA, USA): 0.22 μm pore size for proteins < 20 kDa and 0.45 μm for proteins > 20 kDa, at 400 mA for 8–30 min. Membranes were blocked with 5% bovine serum albumin (BSA) for 1 h at room temperature, followed by overnight incubation with primary antibodies at 4 °C. After washing with TBST, membranes were incubated with HRP-conjugated secondary antibodies for 1 h at room temperature. Protein bands were visualized using enhanced chemiluminescence (ECL) substrate. All electrophoresis and transfer buffers were purchased from Servicebio (Wuhan, China). Antibody details are provided in Table 2.

### 4.9. Transmission Electron Microscopy (TEM)

Cells were fixed in 2.5% glutaraldehyde at 4 °C for 6 h, rinsed twice with PBS (10 min each), and post-fixed in 1% osmium tetroxide for 2 h. After two additional PBS washes, samples were dehydrated through a graded ethanol series, embedded in resin, and ultrathin-sectioned (60–80 nm). Sections were stained with lead citrate and examined under a transmission electron microscope (JEM-2100, JEOL Ltd., Tokyo, Japan).

### 4.10. Intracellular ROS Measurement

Intracellular reactive oxygen species (ROS) levels were measured using a ROS Assay Kit (CAS: S0033S; Beyotime, Shanghai, China). Following the manufacturer’s protocol, nuclei were counterstained with DAPI (CAS: C1002; Beyotime, Shanghai, China). Cells were washed three times with PBS and imaged using a fluorescence microscope. Fluorescence intensity was quantified for statistical analysis.

### 4.11. ATP Assay

Cellular ATP levels were determined using an ATP Assay Kit (CAS: S0027; Beyotime, Shanghai, China). Briefly, 100 μL of detection working solution was added directly to each well of a 6-well plate containing cultured cells, and luminescence was measured according to the kit instructions.

### 4.12. Na^+^-K^+^-ATPase Activity Assay

After sonication of cells in 6-well plates, total protein concentration was quantified using a Coomassie Brilliant Blue Protein Assay Kit (CAS: A045-2; Nanjing Jiancheng Bioengineering Institute, Nanjing, China). Na^+^-K^+^-ATPase activity was then measured using a dedicated assay kit (CAS: A070-2; Nanjing Jiancheng Bioengineering Institute, Nanjing, China), following the manufacturer’s protocol.

### 4.13. Immunofluorescence Staining

Cells were fixed with 4% paraformaldehyde for 20 min, washed three times with PBS (5 min each), and permeabilized with 0.1% Triton X-100 for 15 min. After blocking with 3% BSA for 30 min at 25 °C, cells were incubated overnight at 4 °C with primary antibodies against PINK1 and AIF (or LC3B and TOM20, as indicated). Following three 5 min PBST washes, cells were incubated in the dark for 1 h at room temperature with Cy3- or FITC-conjugated goat anti-rabbit IgG secondary antibodies (Beyotime, Shanghai, China). Nuclei were stained with DAPI, and slides were mounted and imaged using a fluorescence microscope. Images were analyzed using ImageJ software (1.54g) (NIH, Bethesda, MD, USA).

### 4.14. IBR Analysis

Data were standardized using the formula:

$$Y=\frac{\left(X-m\right)}{s}$$

where X is the mean value of a given biomarker in a treatment group, m is the overall mean across all groups, and s is the overall standard deviation. For biomarkers that were upregulated upon treatment, Z=Y; for those that were downregulated, Z=−Y. The IBR score for each biomarker in each group was calculated as:

S=Z+∣min(Z)∣

These scores were plotted as radial lengths in a radar chart. The IBR index for each group was calculated based on the total area enclosed by adjacent biomarker vectors, with a higher IBR value indicating a greater overall toxicological impact. The IBR results facilitated the comparison of toxic effects among different treatment groups and doses, as higher IBR indices suggest more severe cellular damage. Furthermore, these results assist in identifying key biomarkers associated with toxicity by analyzing the radial lengths, thereby providing a foundation for further exploration of the toxic mechanisms of test substances in cytotoxicity experiments.

### 4.15. Statistical Analysis

All experiments were performed with biological triplicates (n = 4/6), and technical repetitions were set in each group. Data were analyzed using GraphPad Prism 9.0 (GraphPad Software, San Diego, CA, USA). One-way ANOVA followed by Tukey’s post hoc test was used to compare differences among multiple groups. Results are expressed as mean ± standard error of the mean (SEM). A *p* < 0.05 was considered statistically significant.

### 4.16. Cell Lines

Human neuroblastoma cells (SH-SY5Y), RRID: CVCL_0019, China Center for Type Culture Collection.

## 5. Conclusions

Exposure to SIM can cause structural damage and dysfunction of mitochondria in SH-SY5Y cells, ultimately inducing apoptosis through inhibiting mitophagy via the *PINK1/Parkin* signaling pathway. Pretreatment with SAL can effectively prevent this injury and aid in protective paralysis. This research provides a new approach in the early prevention and treatment of dopaminergic neuron damage and a new avenue for the development of safe and acceptable natural antagonists in mediating environmental chemical-induced neurotoxicity.

## Figures and Tables

**Table 1:** Primers used for real-time PCR analysis.

Gene	Forward Primer (5′→3′)	Reverse Primer (5′→3′)
*β-actin*	cctggacttcgagcaagagatgg	caggaaggaaggctggaagagtg
*TH*	cgcagttctcgcaggacattg	actccaccgtgaaccagtacag
*LC3B*	acttattcgagagcagcatccaac	acatggtcaggtacaaggaactttg
*TOM20*	gcaggtcttacagcaaactcttcc	tcattccacatcatcttcagccaag
*AIF*	ctacaagcacgctctaacatctgg	cagccaatcttccactcacaacag
*PARP-1*	cagagtatgccaagtccaacagaag	cagcggtcaatcatgcctagc
*PINK1*	cggctggaggagtatctgatagg	aatgtaggcatggtggcttcatac
*P* *arkin*	aagcagcctccaaagaaaccatc	actcgcagccacagttccag

**Table 2:** Primary antibodies used in this study.

Antibody	Catalog	Company
PINK1	NBP2-36488	Novus Biologicals, Littleton, CO, USA
Parkin	66674-1-Ig	Sanying Biotechnology, Wuhan, China
TH	58844S	Cell Signaling Technology, Danvers, MA, USA
LC3B	43566	Cell Signaling Technology, Danvers, MA, USA
AIF	A19536	ABclonal Biotechnology, Wuhan, China
TOM20	A19403	ABclonal Biotechnology, Wuhan, China
PARP-1	A19596	ABclonal Biotechnology, Wuhan, China
β-actin	20536-1-AP	Sanying Biotechnology, Wuhan, China
GAPDH	60004-1-Ig	Sanying Biotechnology, Wuhan, China

## Data Availability

The data set we used is from the GEO database, and downloads can be retrieved in the GEO data set at GSE43490.

## Supplementary Materials

The following supporting information can be downloaded at: https://www.mdpi.com/article/10.3390/ijms27104242/s1.

## Abbreviations

The following abbreviations are used in this manuscript:

AIF	Apoptosis Inducing Factor
ATP	Adenosine Triphosphate
cDNA	complementary DNA
LC-3B	Microtubule-associated protein 1 light chain 3 beta
MPP^+^	1-methyl-4-phenylpyridiniumion
Parkin	Parkin RBR E3 ubiquitin protein ligase
PARP-1	Poly (ADP-ribose) polymerase 1
PD	Parkinson’s disease
PINK1	PTEN induced putative kinase 1
ROS	Reactive Oxygen Species
SAL	Salidroside
SIM	Simazine
TH	Tyrosine hydroxylase
TOM20	Translocase Of Outer Mitochondrial Membrane 20
