# Supplementary material for: Salidroside Protects Against Simazine-Induced Neurotoxicity by Activating PINK1/Parkin Mitophagy
Source: Int J Mol Sci. 2026 May 10;27(10):4242. doi: 10.3390/ijms27104242 (PMC13206927; doi:10.3390/ijms27104242)
Supplement: Supplementary file 1 [file ijms-27-04242-s001.zip › ijms-4227313-supplementary.pdf]

Salidroside Protects Against Simazine -Induced Neurotoxicity by Activating *PINK1*/*Parkin* Mitophagy

Xueting Li [1,2], Yi Xiang [1], Jiaqi Li [1], Hewei Song [1], Chunlong Zhao [1], Baixiang Li [1,2*]

1 Department of Hygienic Toxicology, School of Public Health, Harbin Medical University, Harbin 150081, China; lxting@hrbmu.edu.cn (X.L.); 2024020249@hrbmu.edu.cn (Y.X.); 2024020205@hrbmu.edu.cn (J.L.); 2024020210@hrbmu.edu.cn (H.S.); 2024020203@hrbmu.edu.cn (C.Z.)
2 Key Laboratory of Precision Nutrition and Health, Ministry of Education, Harbin Medical University, Harbin 150081, China
* Correspondence: libaix@ems.hrbmu.edu.cn

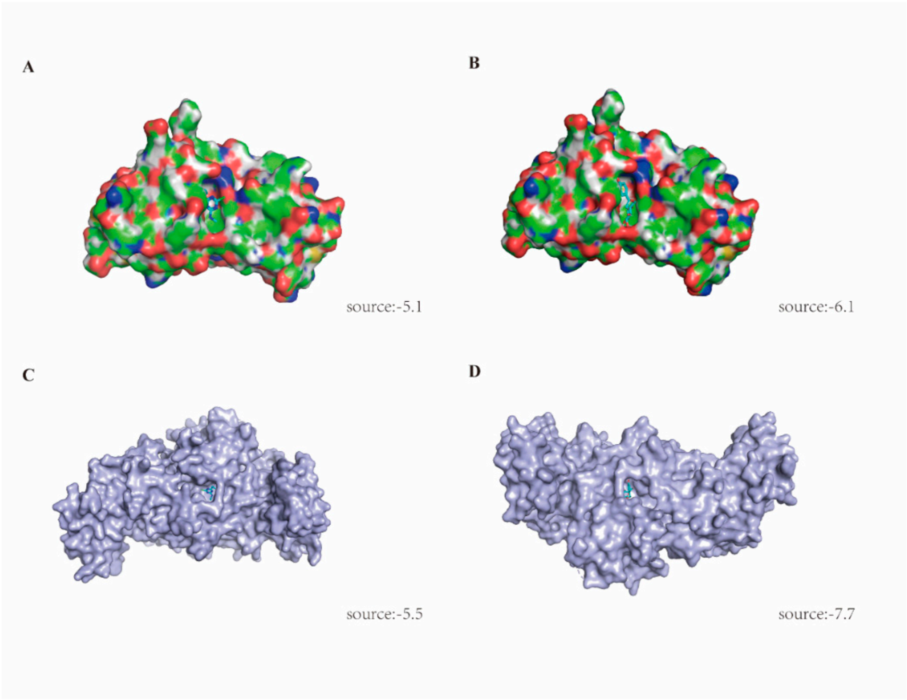

Figure S1 Structural visualization of Parkin/PINK1-ligand complexes.

Panels correspond to: (A) Parkin–SIM, (B) Parkin–SAL, (C) PINK1–SIM, and (D) PINK1–SAL.

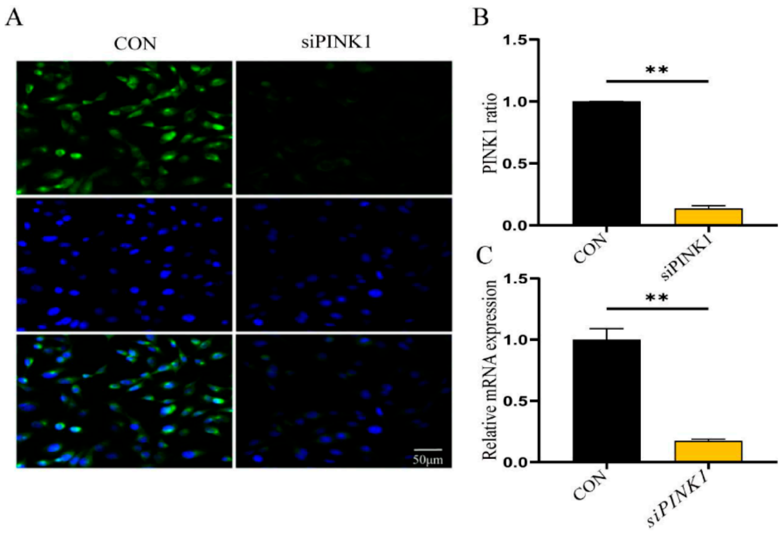

Figure S2 Changes in PINK1 fluorescence intensity and mRNA expression levels after.
